# How far are we off? Analyzing the accuracy of surgical margin relocation in the head and neck

**DOI:** 10.1002/hed.27793

**Published:** 2024-05-04

**Authors:** Alexis Miller, Vickie Wang, Victor Jegede, Fabian Necker, Joseph Curry, Fred M. Baik, Avanti Verma, F. Christopher Holsinger, Madalina Tuluc, Mobeen Rahman, James S. Lewis, Eben Rosenthal, Michael C. Topf

**Affiliations:** 1 Department of Otolaryngology – Head and Neck Surgery, Vanderbilt University Medical Center, Nashville, Tennessee, USA; 2 Division of Otolaryngology, Department of Surgery, Yale School of Medicine, New Haven, Connecticut, USA; 3 Lewis Katz School of Medicine, Temple University, Philadelphia, Pennsylvania, USA; 4 Institute of Functional and Clinical Anatomy, Friedrich-Alexander University Erlangen-Nürnberg, Erlangen, Germany; 5 Department of Otolaryngology – Head & Neck Surgery, Thomas Jefferson University Hospital, Philadelphia, Pennsylvania, USA; 6 Department of Otolaryngology – Head and Neck Surgery, Stanford University, Palo Alto, California, USA; 7 Department of Pathology, Thomas Jefferson University Hospital, Philadelphia, Pennsylvania, USA; 8 Department of Pathology, Stanford University, Palo Alto, California, USA; 9 Department of Pathology, Microbiology, and Immunology, Vanderbilt University Medical Center, Nashville, Tennessee, USA

**Keywords:** head and neck cancer, margin relocation, re-resection, surgical margins

## Abstract

**Background:**

Positive surgical margin rates remain high in head and neck cancer surgery. Relocation is challenging given the complex, three-dimensional (3D) anatomy.

**Methods:**

Prospective, multi-institutional study to determine accuracy of head and neck surgeons and pathologists relocating margins on virtual 3D specimen models using written descriptions from pathology reports. Using 3D models of 10 head and neck surgical specimens, each participant relocated 20 mucosal margins (10 perpendicular, 10 shave).

**Results:**

A total of 32 participants, 23 surgeons and 9 pathologists, marked 640 margins. Of the 320 marked perpendicular margins, 49.7% were greater than 1 centimeter from the true margin with a mean relocation error of 10.2 mm. Marked shave margins overlapped with the true margin a mean 54% of the time, with no overlap in 44 of 320 (13.8%) shave margins.

**Conclusions:**

Surgical margin relocation is imprecise and challenging even for experienced surgeons and pathologists. New communication technologies are needed.

## 1 | INTRODUCTION

Surgical margin status is an important prognostic factor in head and neck squamous cell carcinoma (HNSCC), impacting both local recurrence and overall survival.^1,2^ Unfortunately, HNSCC has the highest rates of positive surgical margins of all solid malignancies affecting both men and women,^3^ estimated to occur at a rate of 18% across all anatomic subsites.^4^ Compared to tumor- and patient-related prognostic factors, surgical margin status is potentially modifiable through communication between surgeons and pathologists.

When encountered, a positive surgical margin is re-resected if feasible.^5^ However, several studies have demonstrated that re-resecting to negative margins does not significantly improve local recurrence rates or survival outcomes for patients.^6–9^ Margin relocation in the head and neck is particularly challenging given the three-dimensional (3D) anatomic complexity of the region and variety of tissue subtypes often represented in specimens.^10^ Only an estimated one third of re-resection specimens contain additional carcinoma following a cut-through margin, suggesting that surgeons may have difficulty accurately relocating margins during re-resection.^11^

Over 20 years ago, a study analyzing margin relocation accuracy showed over 30% of relocations exceeded 1 cm from the initial margin site.^12^ Our study seeks to expand upon these previous studies and further evaluate the accuracy of margin relocation in HNSCC. Utilizing our previously developed protocol for 3D scanning and virtual 3D specimen mapping^13,14^ as well as the corresponding written pathology reports, we aim to quantify the accuracy of surgical margin relocation by expert-level surgeons and pathologists using a novel method for quantitative analysis of margin location.

## 2 | MATERIALS AND METHODS

### 2.1 | Study design and participants

This multi-institutional prospective study was approved by the Vanderbilt Institutional Review Board (#221609), and consent was obtained from all participants. A total of 10 previously 3D scanned and virtually mapped head and neck oncologic specimens were included in the study. Specimens were 3D scanned and virtually mapped according to the team’s previously described protocols.^13,14^ The specimens consisted of eight oral cavity composite resections and two lateral oropharyngectomies. Two mucosal margins from each specimen were selected, representing a variety of mucosal locations on the specimens and evenly distributed between perpendicular (10) and shave (10) margins for the entire study. There was no limit to the length of shave margins selected for this study. Deep margins were not assessed in this study. The written specimen description, margin labels, and margin descriptions were extracted from the actual pathology report for each case. Representative screenshots of the 10 virtual 3D specimen models with the actual shave and perpendicular margins annotated (“true margins”) are available in the supplement (Table S1).

All participant enrollment and data collection were performed between March and June 2023. Only attending or fellow head and neck surgeons and pathologists were recruited to participate in this study. Surgeons and pathologists involved in study design were excluded from participation. A pre-study power calculation determined that with an estimated SD of 5.7 mm based on a previous relocation study^15^ at least 31 participants would provide 90% power to detect a 2 mm difference between margins with a two-tailed 95% confidence interval (CI).

**FIGURE 1:**
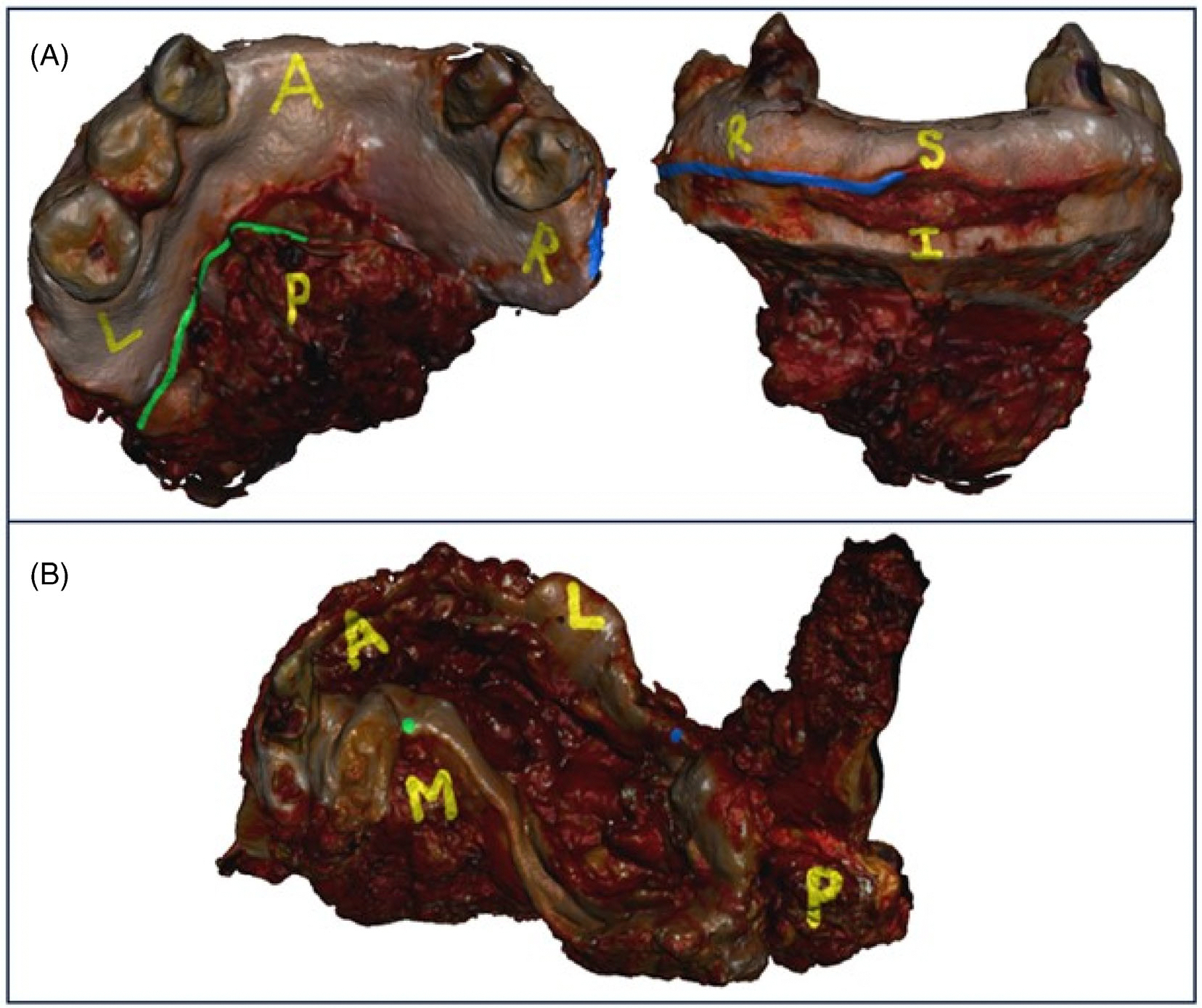
Virtual 3D specimen model with descriptions from final pathology report. Virtual 3D model with anatomic orientation labels in yellow. Participants were oriented to the specimen by a trained research assistant and were able to rotate the virtual model before marking two margins on each specimen. Shown here (A) is an “oral cavity composite resection of the anterior alveolar ridge and marginal mandibulectomy” with “left superficial/superior mucosal margin, shave” (green) and “right anterior mucosal margin, shave” (blue). The “medial floor of mouth margin, perpendicular” (green) and “lateral retromolar trigone margin, perpendicular” (blue) are shown on “right oral cavity composite resection with hemi-mandibulectomy” (B). [Color figure can be viewed at wileyonlinelibrary.com]

During in-person study administration, participants were shown an instructional video (Video S1), including study overview and brief tutorial, and then asked to relocate the margins on each specimen in computer-aided design (CAD) software (Meshmixer, Autodesk Inc., San Rafael, CA). The participant was able to rotate and zoom in and out on the 3D model in the CAD software. Participants were oriented to each virtual 3D specimen model by a trained study coordinator (A.M., V.W., V.J.) using standardized anatomic orientation labels and the specimen description from the final pathology report (Figure 1). The trained study coordinator was immediately available throughout the entire study. The written margin description from the final pathology report was read, and the participant relocated and virtually annotated the site of the margin to the best of their ability. A written list of all margins (not just the two margins selected for the study) sectioned from the specimen was also available to the participant for reference. This process was repeated for all 10 specimens with a total of 20 surgical margins. Perpendicular margins were annotated as a dot on the mucosal edge and shave margins as a line along the mucosal edge. There was no limit to the length that could be marked for shave margins.

### 2.2 | Quantitative and statistical analysis

**FIGURE 2:**
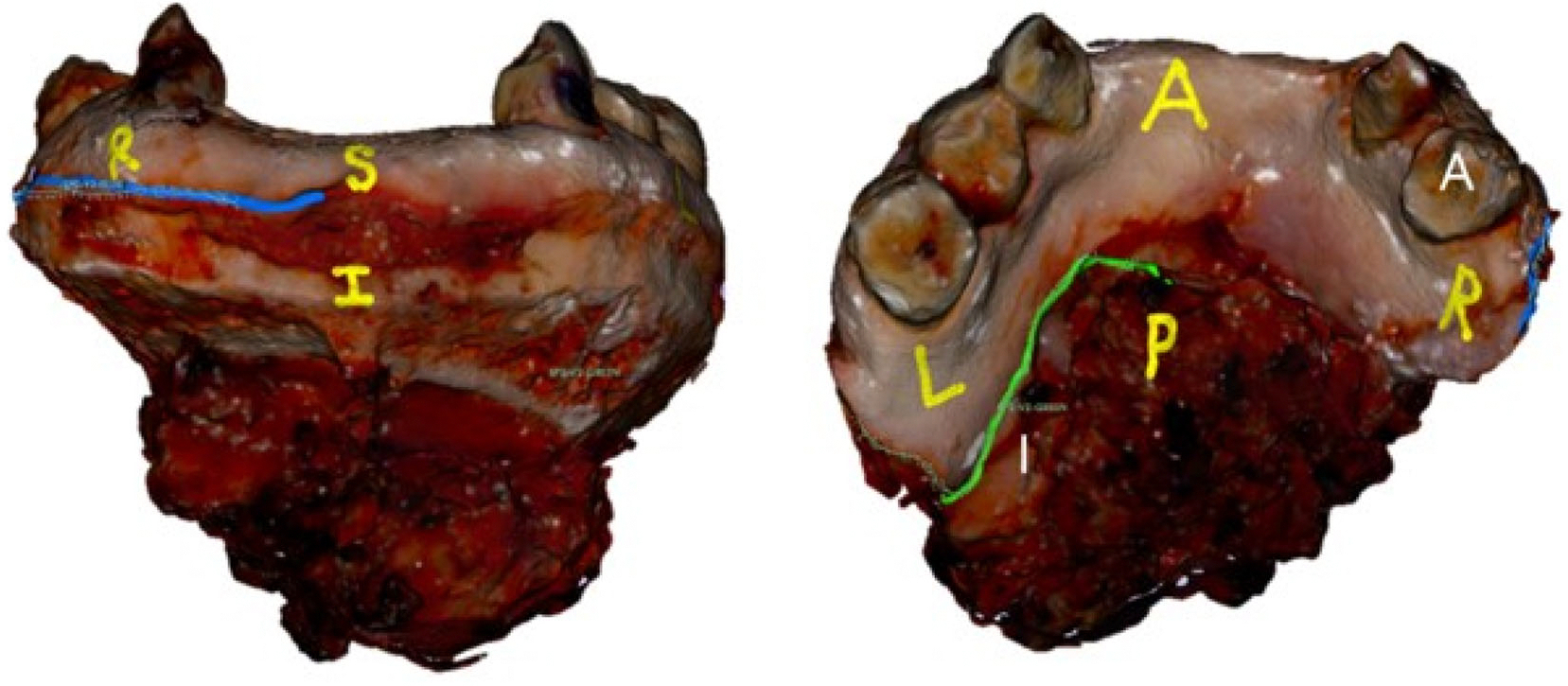
Virtual 3D specimen model with true and participant-marked margin shown for analysis in 3D Slicer. The true shave margins are shown as solid green and blue lines. The dashed 100-point line with a corresponding label represents the margin marked by the study participant. These participant-marked margins were compared to the true shave margin using 100-point analysis in 3D Slicer. [Color figure can be viewed at wileyonlinelibrary.com]

To analyze the accuracy of margin relocation, participant-marked margins were compared to the true margin which was marked onto the virtual 3D model alongside the prosector during specimen processing as previously described.^14^ The distance between the participant-marked and true margin was measured for perpendicular margins. For shave margins the percent overlap between participant-marked and true margin (within 2 mm) was measured by matching the location of 100 points on each shave margin (Figure 2), starting from the middle point on the participant margin and matching to the closest point on the true margin. The 100-point matching approach allows for participants to be equally penalized for over-estimating or under-estimating the length of the shave margin. All quantitative margin analysis was performed using 3D Slicer (version 5.2.2, Boston, MA).^16^

Descriptive statistics were utilized to summarize the data, and Wilcoxon signed-rank test was performed to compare margin relocation between surgeons and pathologists. Statistical significance was defined as *p* value less than 0.05. All analyses were performed in R Studio (version 2023.06.1+524; packages: ggplot2, tidyr, dplyr, stringr, readxl, openxlsx; Vienna, Austria).

## 3 | RESULTS

A total of 32 physicians participated in this study, consisting of 23 (72%) head and neck surgeons and 9 (28%) pathologists. Surgeon participants were mostly professors (*n* = 8, 35%) and associate professors (*n* = 7, 30%), while assistant professors (*n* = 4, 17%), and clinical instructors (*n* = 4, 17%) were represented less often. Pathologists were most often full professors (*n* = 4, 44.4%), followed by assistant professors (*n* = 2, 22.2%), fellows (*n* = 2, 22.2%), and associate professors (*n* = 1, 11.1%). Participants were well-distributed across the 4 participating institutions, ranging from 6 to 11 participants from each institution. In total, 640 margins were marked, evenly distributed with 320 (50%) shave margins, and 320 (50%) perpendicular margins, across the 10 surgical specimens.

**FIGURE 3:**
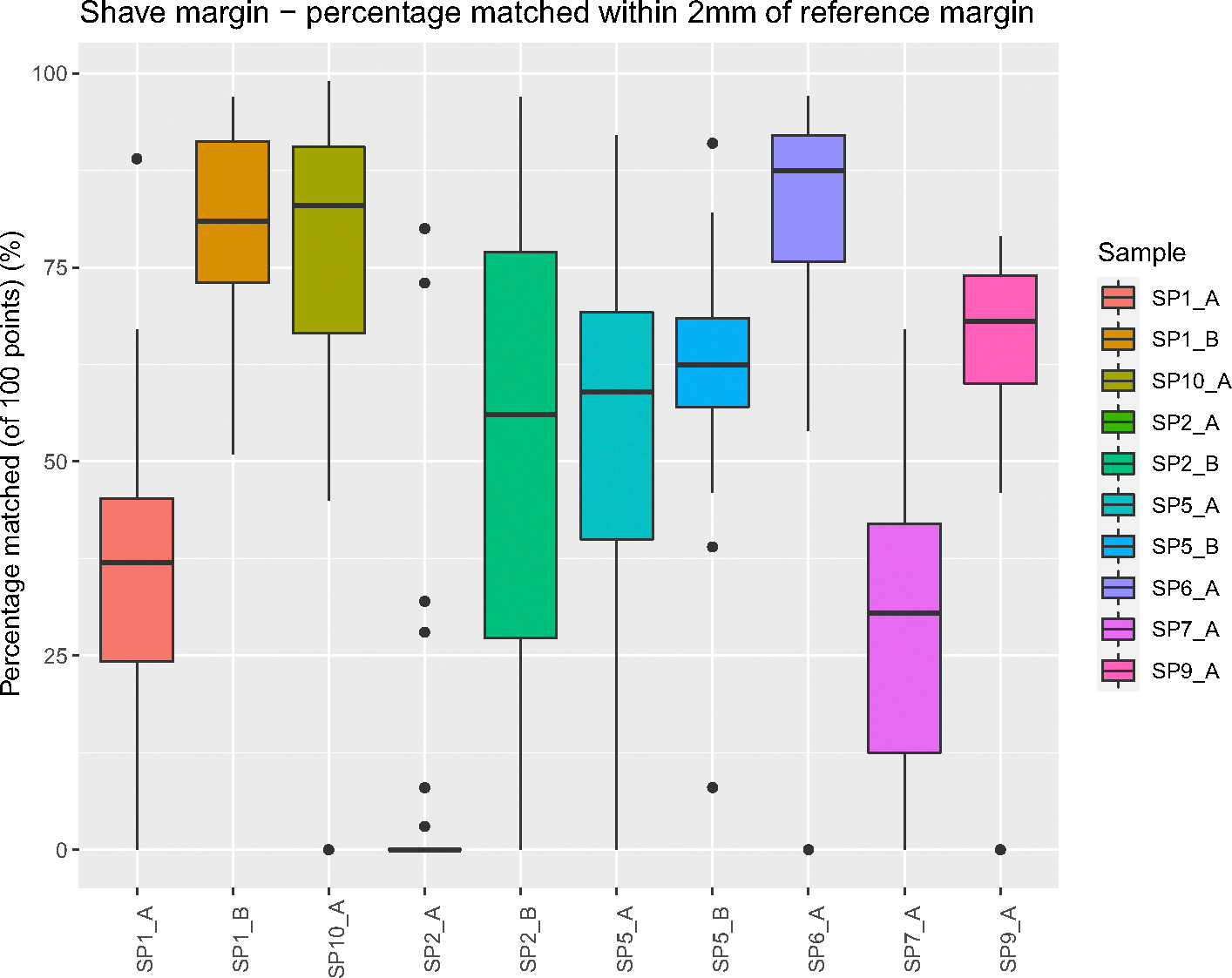
Relocation error of participant-marked shave margins. For each of the 10 shave margins in this study, the percent overlap between the margins marked by participants and the true margin is shown. A smaller percentage of overlap indicates poorer relocation accuracy. [Color figure can be viewed at wileyonlinelibrary.com]

The participant-marked shave margins had a mean (SD; range) overlap of the true margin of 54% (30.75%; 0%–99%) with a median (IQR) of 62% (45.25%). Of the 320 shave margins marked, 44 (13.75%) had 0% overlap with the true margins. A total of 37% (118/320) of shave margins had less than 50% overlap with the true shave margin. The overlap varied between each margin (Figure 3) with the least accurately relocated shave margin from an oral cavity composite resection specimen (Specimen 2, Margin A; Table S1) with a mean (SD; range) overlap of 7% (19.71%; 0%–80%). Two shave margins, one from a buccal resection (Specimen 1, Margin B) and the other from an oral cavity composite resection with marginal mandibulectomy (Specimen 6, Margin A), were most accurately relocated with a mean (SD; range) overlap of 81% (11.66%; 51%–97%) and 81% (19.41%; 0%–97%).

**FIGURE 4:**
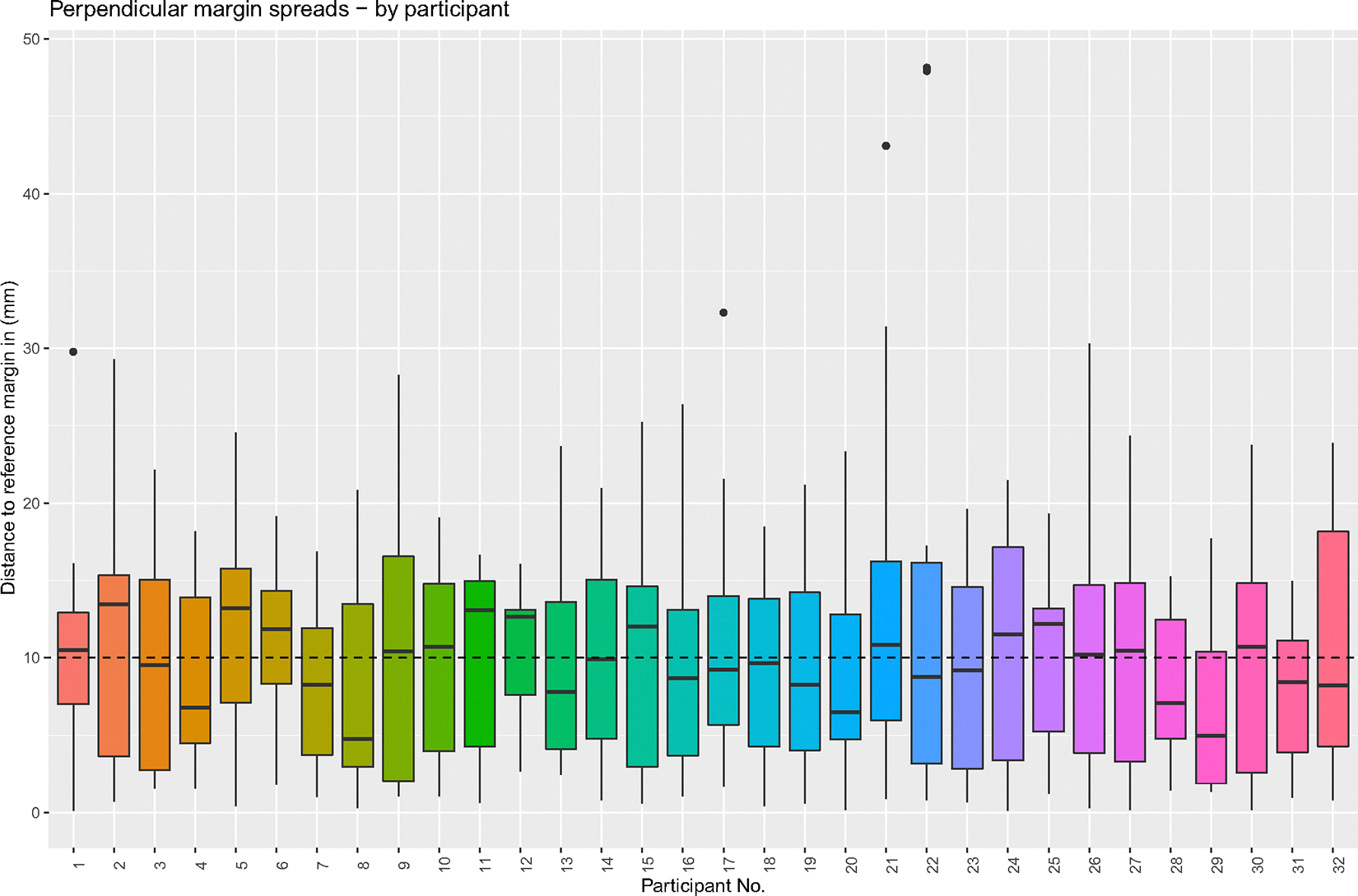
Distance from participant-marked to true perpendicular margin for each participant. Each participant is represented on the x-axis, and the distance from the participant-marked margin to the true margin is represented on the y-axis. For each participant, the data for all 10 perpendicular margins is represented. The dotted line represents the average distance from the true margin (10.22 mm). [Color figure can be viewed at wileyonlinelibrary.com]

Participant-marked perpendicular margins were on average (SD; range) 10.2 mm (7.83 mm; 0.1–48.1 mm) from the true margin with a median (IQR) distance of 9.8 mm (11.0 mm) (Figure 4). Of the 320 perpendicular margins marked, only 43 (13.4%) were less than 2 mm from the true margin, and 34% (*n* = 109) were within 5 mm. Meanwhile, 50% (*n* = 159) of the perpendicular margins were greater than 1 cm from the true margin with 9% (*n* = 29) more than 2 cm away. The most accurately relocated perpendicular margin, from an oral cavity composite resection with marginal mandibulectomy (Specimen 3, Margin B), was an average (SD; range) distance of approximately 1 mm (0.74 mm; 0.11–2.64 mm). Meanwhile, the least accurate perpendicular margin, on an oral cavity composite resection with hemi-mandibulectomy (Specimen 4, Margin A), was an average (SD; range) of almost 19 mm (11.33 mm; 1.28–47.92 mm) from the true margin location.

**FIGURE 5:**
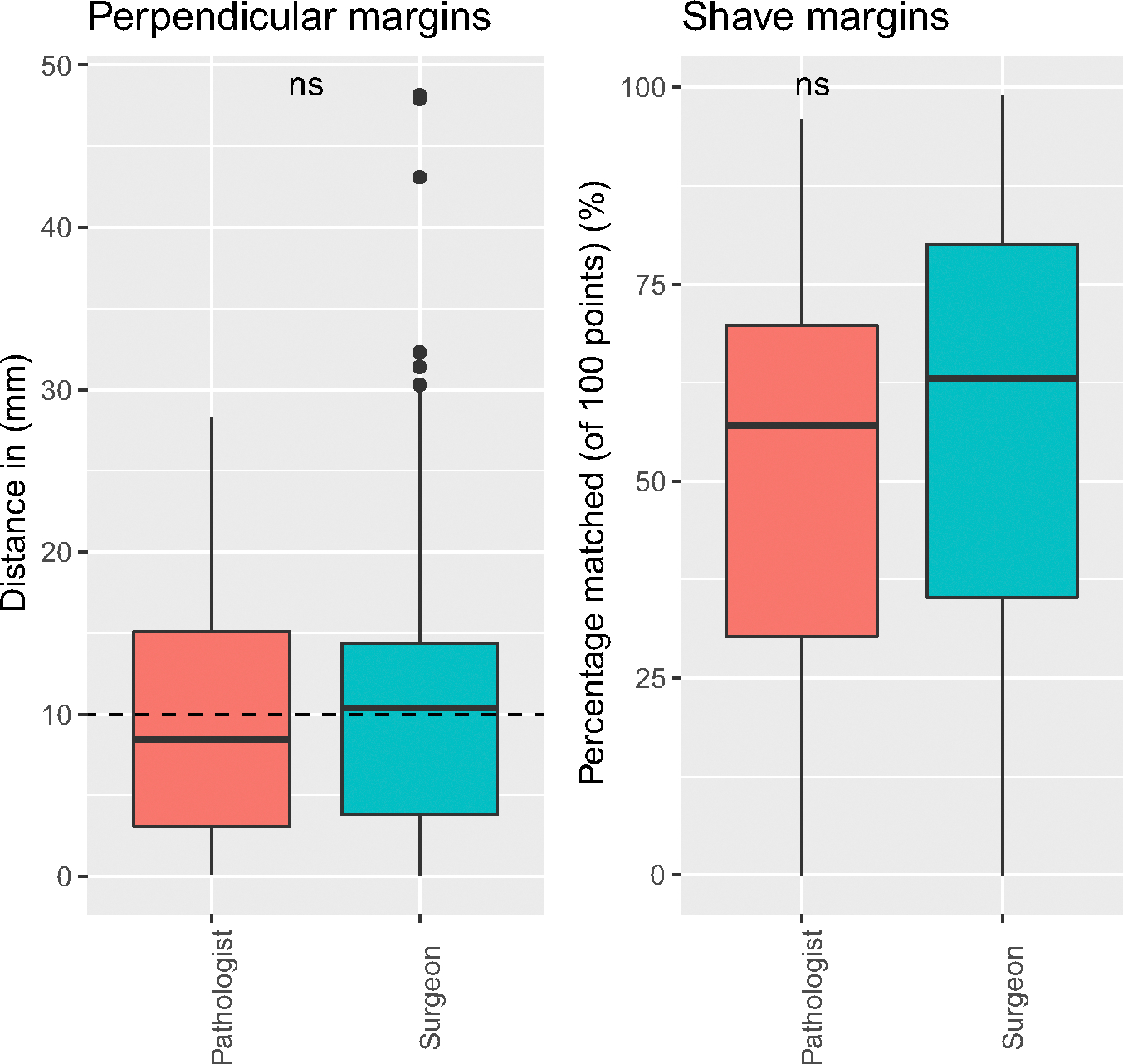
Comparison of margin relocation accuracy between surgeons and pathologists for perpendicular and shave margins, respectively. [Color figure can be viewed at wileyonlinelibrary.com]

There was no significant difference between surgeons and pathologists in relocation of shave (*p* = 0.57) or perpendicular margins (*p* = 0.13) (Figure 5).

## 4 | DISCUSSION

This study evaluated the accuracy of margin relocation by expert-level head and neck surgeons and pathologists and confirms that in this 3D specimen model study relocation of surgical margins is imprecise. To our knowledge, this is the first study to evaluate the relocation accuracy of shave margins. When asked to relocate a shave margin, participants missed 50% or more of the margin 37% of the time. When asked to identify a perpendicular margin, participants were, on average, 10.2 mm away from the true margin location.

Surgical margin status is the most important prognostic factor for patients with head and neck cancer undergoing surgical resection.^1,2,17,18^ Unlike many patient and tumor prognostic factors, margin status is the only variable that can be impacted by surgeons and pathologists. However, there continues to be a lack of consensus about surgical margins. A recent survey study of American Head and Neck Society (AHNS) members identified dissensus in defining a negative margin and preferred margin sampling techniques.^19^ This study also found that 81% of surgeons considered a positive margin cleared to negative via frozen section analysis to be equivalent to a negative margin.^19^ However, prior studies have shown that re-resection to a negative margin on final pathology does not improve recurrence rates compared to patients with a final positive margin.^6–9^ This is unsurprising given that only 20%–29% of re-resection specimens contain additional malignancy, suggesting that surgeons may have difficulty accurately relocating surgical margins.^11,20^ Our study results support these findings as relocation error exceeded 1 cm for nearly half (49.7%) of perpendicular margins, suggesting that re-excision of additional tissue may miss residual malignancy in a substantial number of cases.

In the present study, we report that surgeons and pathologists are similarly prone to the challenges of accurate margin relocation. This highlights the challenges of both intraoperative and postoperative communication of margin sampling sites between surgeons and pathologists. However, in the previously mentioned study by Banoub et al., they reported that surgeons and pathologists differed in their ability to localize margins.^15^ Pathologists had greater variability than surgeons with mean relocation error of 12.6 and 21.2 mm, respectively. Despite the prognostic importance of positive surgical margins, there has been limited investigation on the accuracy of margin relocation. Consistent with our result, a single surgeon study reported an average margin relocation error of 9 mm for mucosal and 12 mm for deep margins with over 30% of margins exceeding 1 cm from the initial margin sampling site.^12^ A more recent study evaluated margin relocation using preoperative radiographic 3D models and reported a mean error of 12.6 mm for surgeons locating margins based on the anatomic descriptions.^15^ These prior studies are consistent with our study which identified a mean relocation error of 10.2 mm.

Taken together, these studies demonstrate the importance of leveraging novel technologies to provide a permanent visual record of the specimen and the sites of margin sampling rather than relying on written descriptions.^14^ One can envision that the pathology report of the future will be a mixed-media report including a virtually-mapped 3D specimen model to aid in post-operative communication of margin status between surgeons and pathologists.^21^ These innovations highlight the importance of information hand-off between members of the interdisciplinary cancer care team as a potential site of error that could be targeted for improvement. In an effort to improve margin relocation on the specimen, recent studies describe the use of 3D scanning with video MILLER conference call for intraoperative communication of frozen section results.^13,22,23^ To address relocation related to the surgical bed, a recent study detailed intraoperative 3D scanning of the surgical defect,^24^ while we have investigated use of augmented reality to replace annotated virtual 3D specimen models back into the surgical defect.^25^ While the current study focused on relocation onto the specimen itself to identify challenges in communication between surgeons and pathologists regarding sites of margin sampling, the authors recognize that relocating the margin in the tumor bed may be more clinically relevant. Unfortunately, the current quality of tumor bed 3D scans prohibited our team from investigating relocation within the tumor bed.

Without strategies to mitigate the challenges associated with surgical margin relocation, it will continue to impact postoperative oncologic care including adjuvant radiation therapy planning. We are currently investigating the utility of these 3D virtual specimen maps^14^ in adjuvant radiation planning (NCT #05743569). We hypothesize that radiation oncologists will benefit from a visual reference for sites of margin sampling, possibly allowing for more targeted adjuvant radiation. Future efforts to integrate technologic advancements into surgical margin relocation should be evaluated to potentially improve patient outcomes and prove value.

There are several limitations to this study. The major identified limitation is that the study participants were not clinically involved in the study cases. However, specimens were adequately anatomically oriented using standardized labels and via instruction by the trained research assistant who was available during the entire study. Each of three research assistants engaged in over 5 h of training prior to administering the study to participants. A standardized script was used during study administration to maximize the reliability of the results across all institutions. The utilized technology, including quality of virtual 3D scans and the CAD software for annotation, is another possible limitation. Research assistants clarified understanding of the tissue types present on the 3D model as well as location of the mucosal edge. Since this information was provided during study administration, participants were not penalized for approximation to the mucosal edge during analysis. The true margin used as reference in this study was documented during 3D virtual specimen mapping alongside the pathology prosector which requires a trained research member to accurately record the margin location. At our institution, margin sections and labels are typically selected by a trained pathologists’ assistants (PA’s), pathologist, or surgeon depending on the case.

The specificity of anatomic descriptions provided in the margin label may impact the ability of the surgeon to relocate. We recognize that the results of this study are potentially dependent on the margins descriptions used by pathologists in their pathology reports. It is possible that more descriptive margin labels would have resulted in less relocation error in this study. However, we wanted to use the actual margin labels corresponding with the 3D cases. Regardless, more descriptive, anatomic margin labeling should be considered to improve communication among the multidisciplinary care team.

Margins used in this study were sampled both intraoperatively, as frozen sections during a specimen-based approach, and postoperatively on final pathologic sectioning. It is worth noting that there is variability in margin sampling techniques on an institutional and even an individual level, and this may contribute to the variability in margin relocation. Deep margins were not included in this study due to concern for increased difficulty with nuanced anatomic landmarks needed for relocating deep margins using the virtual 3D specimen models. While the current study focused on mucosal margins, deep margins are more frequently involved in positive margin cases.^26^ Both Kerawala et al. and Banoub et al. identified worse relocation of deep margins compared to mucosal margins, suggesting that the location of deep margins is more challenging to accurately relocate.^12,15^ This is supported by evidence of poorer survival outcomes with a deep initial positive margin compared to mucosal regardless of re-resection.^20^ Future expansion of this study to include deep margins is warranted to further investigate this relationship. Additionally, this study utilized 10 head and neck surgical cases with the majority being oral cavity resections with mandibulectomy. Further studies should expand upon this limited number and variety of cases to further increase applicability of the findings.

## 5 | CONCLUSION

Relocation of surgical margins is imprecise. The distance from the margin location exceeded 1 cm for nearly half of the perpendicular margins. Shave margins failed to overlap with greater than half of the true margin in over one third of cases. Technologic advancements and enhanced intraoperative communication are needed to improve reliable surgical margin relocation.

## Supplementary Material

Supplemental Table

Supplemental Instructional Video

## Data Availability

The data that support the findings of this study are available from the corresponding author upon reasonable request.
