# Supplementary material for: How far are we off? Analyzing the accuracy of surgical margin relocation in the head and neck
Source: Head Neck. Author manuscript; Available in PMC 2025 Apr 26. (PMC12032842; doi:10.1002/hed.27793)
Supplement: Supplemental Table [file NIHMS2071327-supplement-Supplemental_Table.docx]

**SUPPLEMENTAL MATERIAL**

Table 1. Summary of Specimens and Margins in Study

**Table1. Summary of Specimens and Margins in Study**

| **Specimen No.** | **Specimen Description** | **Margin Descriptions** | **Virtual 3D Model with True Margins Annotated (A=green, B=blue)** |
| --- | --- | --- | --- |
| 1 | Right buccal mucosa resection with overlying skin | A) Anterior mucosal margin, shave  B) Superior mucosal margin, shave | 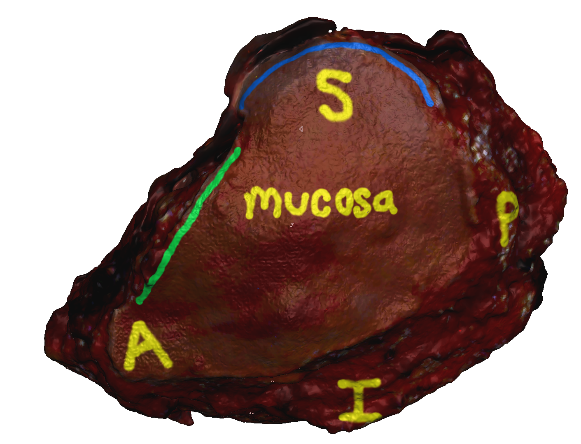 |
| 2 | Oral cavity composite resection of the anterior alveolar ridge and marginal mandibulectomy | A) Left superficial/superior mucosal margin, shave  B) Right anterior mucosal margin, shave | 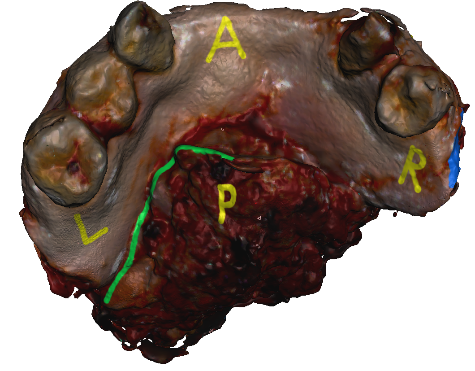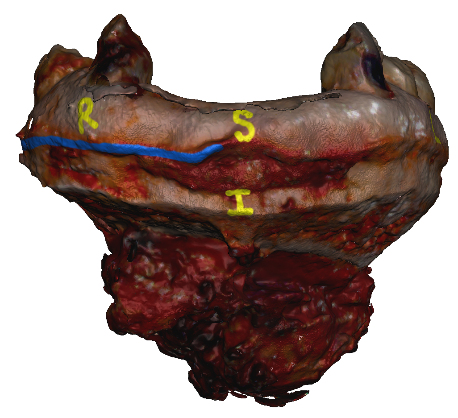 |
| 3 | Oral cavity composite resection of the anterior floor of mouth with marginal mandibulectomy | A) Mass to posterior resection margin, perpendicular  B) Mass to mandible including anterior margin and inferior bone margin, perpendicular | 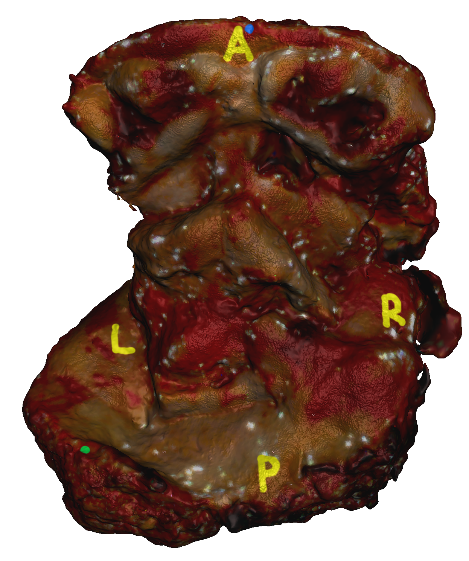 |
| 4 | Right oral cavity composite resection with hemi-mandibulectomy | A) Medial floor of mouth margin, perpendicular  B) Lateral retromolar trigone margin, perpendicular | 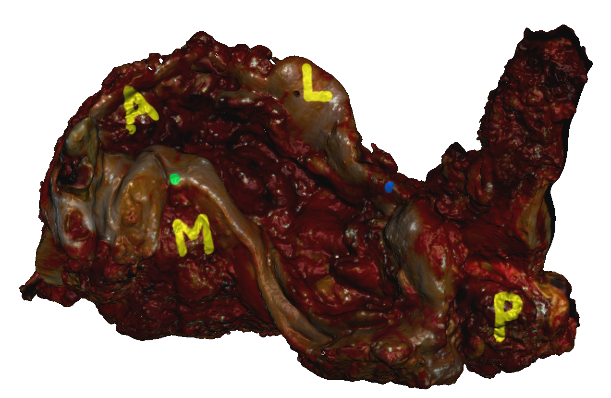 |
| 5 | Oral cavity composite resection of the left mandible with segmental mandibulectomy | A) Anterior lateral mucosa, shave  B) Posterior medial mucosa, shave | 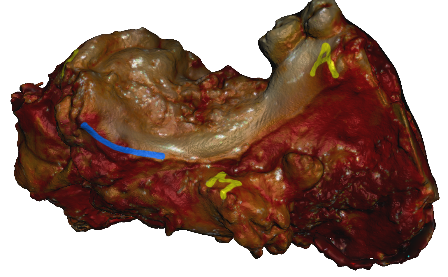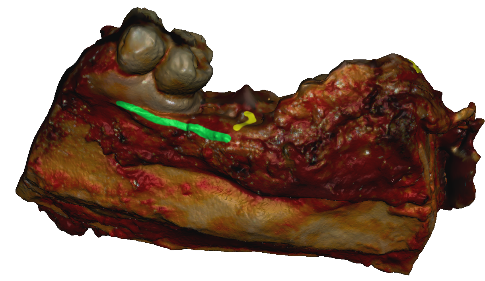 |
| 6 | Left oral cavity composite resection including tongue and marginal mandibulectomy | A) Lateral mucosal margin, shave  B) Mass to medial margin, perpendicular | 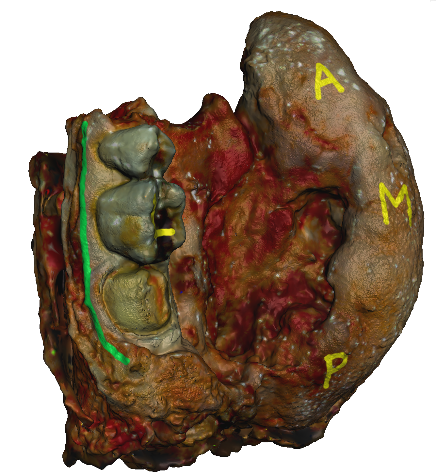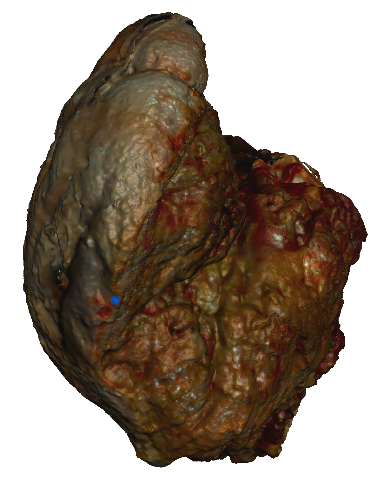 |

| **Specimen No.** | **Specimen Description** | **Margin Descriptions** | **Virtual 3D Model with True Margins Annotated (A=green, B=blue)** |
| --- | --- | --- | --- |
| 7 | Left oral cavity composite resection with marginal mandibulectomy | A) Anterior mucosal margin, shave  B) Lesion to lateral margin, perpendicular | 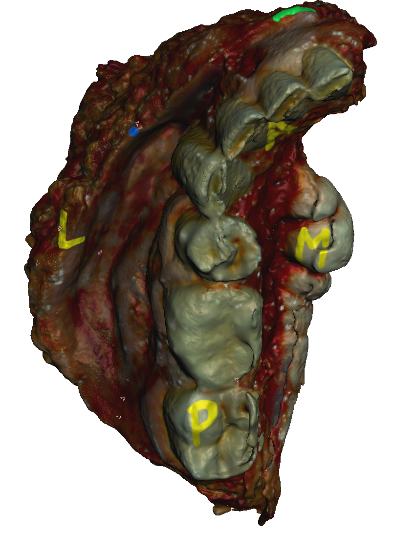 |
| 8 | Left radical tonsillectomy | A) Mass to inferior margin, perpendicular  B) Mass to anterior margin, perpendicular | 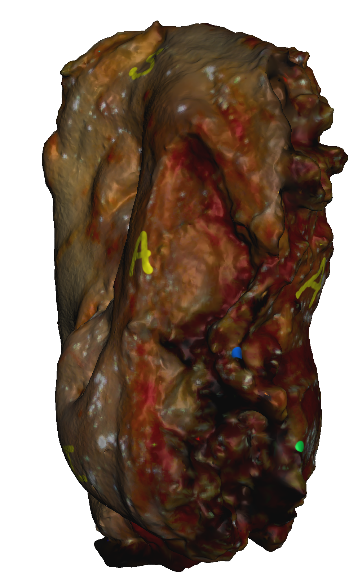 |
| 9 | Anterior oral cavity composite resection with segmental mandibulectomy | A) Right lateral margin, shave  B) Right anterior margin, perpendicular | 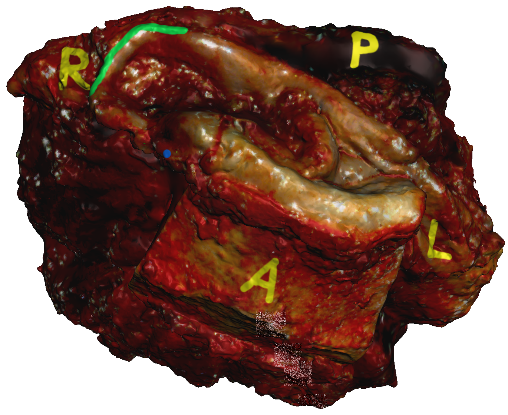 |
| 10 | Left radical tonsillectomy | A) Anterior margin, shave  B) Inferior margin, perpendicular | 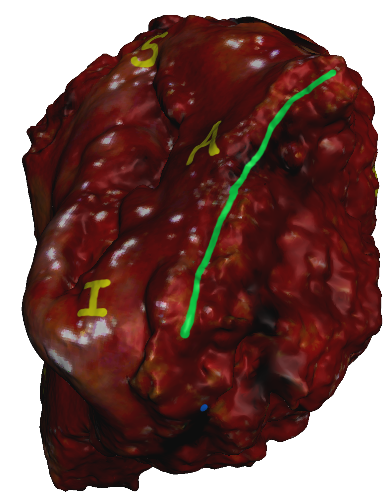 |
